# Position-Sensitive Silicon Photomultiplier Arrays with Large-Area and Sub-Millimeter Resolution

**DOI:** 10.3390/s24144507

**Published:** 2024-07-12

**Authors:** Fabio Acerbi, Stefano Merzi, Alberto Gola

**Affiliations:** Fondazione Bruno Kessler (FBK), Via Sommarive 18, I-38123 Trento, Italy; smerzi@fbk.eu (S.M.); gola@fbk.eu (A.G.)

**Keywords:** silicon photomultiplier, SiPM, single photon, SPAD, gamma ray, position sensitivity

## Abstract

Silicon photomultipliers (SiPMs) are solid-state single-photon-sensitive detectors that show excellent performance in a wide range of applications. In FBK (Trento, Italy), we developed a position-sensitive SiPM technology, called “linearly graded” (LG-SiPM), which is based on an avalanche-current weighted-partitioning approach. It shows position reconstruction resolution below 250 μm on an 8 × 8 mm^2^ device area with four readout channels and minimal distortions. A recent development in terms of LG-SIPM is a larger chip version (10 × 10 mm^2^) based on FBK NUV-HD technology (near-ultraviolet sensitive), with a peak photon detection efficiency at 420 nm. Such a large-area detector with position sensitivity is very interesting in applications like MR-compatible PET, high-energy physics experiments, and readout of time-projection chambers, gamma and beta cameras, or scintillating fibers, with a reduced number of channels. These SiPMs were characterized in terms of noise, photon detection efficiency, and position resolution. We also developed tiles of 2 × 2 and 3 × 3 LG-SiPMs, reaching very large sensitive areas of 20 × 20 mm^2^ and 30 × 30 mm^2^. We implemented a “smart-channel” configuration, which allowed us to have just six output channels for the 2 × 2 elements and eight channels for the 3 × 3 element tiles, preserving a position resolution below 0.5 mm. These kinds of detectors provide a great advantage in compact and low-power applications by maintaining position sensitivity over large areas with a small number of channels.

## 1. Introduction

Silicon photomultipliers (SiPMs) are single-photon-sensitive detectors that continue to attract increasing interest in several industrial and scientific applications that require fast detection, high (single-photon) sensitivity, compactness, insensitivity to magnetic fields, and low bias voltages [1,2,3].

SiPMs are arrays of single-photon avalanche diodes (SPADs) connected in parallel, each one with its own quenching resistor in series, and they output a signal proportional to the number of firing SPADs and, in turn, of detected photons [1]. They usually have an active area between 1 × 1 mm^2^ and 10 × 10 mm^2^ and microcells (i.e., SPADs) with a pitch between few micrometers up to tens of micrometers [1]. SPADs composing the SiPMs work in Geiger mode, being sensitive to single photons. The internal multiplication gain of the single microcells of SiPMs is in the order of a few millions (carriers generated in response to a single photogenerated electron–hole pair) [1].

SiPMs have been employed in a growing number of applications thanks to properties such as compactness, low power consumption, high photon detection efficiency, good time resolution and optical dynamic range. They are currently the detector of choice in many scientific experiments and industrial applications, like Positron emission tomography, with time-of-flight information (ToF-PET) [3,4], Cherenkov light readout [5,6], liquid noble gases scintillators readout at cryogenic temperatures [7,8] and in industrial and automotive light detection and ranging (LiDAR) systems [9]. They are also quickly replacing photomultiplier tubes (PMTs) or hybrid photodiodes (HPDs) in high-energy physics (HEP) experiments (like CMS, LHCb, etc.) [10] and for the readout of scintillators in gamma-ray detectors for space experiments [11,12].

In FBK (Trento, Italy), we developed different SiPM and SPAD technologies, optimized for various applications [13]. The main development branches are, for example, NUV-HD technology (sensitive in the near ultraviolet, with detection efficiency typically peaked at 420 nm) [14], RGB-HD technology (“Red Green Blue”, with sensitivity peaked at green wavelength) [15], NIR-HD (sensitivity extended toward the near-infrared region) [16], and VUV-HD (improved sensitivity for the vacuum ultraviolet) [17]. Such technologies are based on different silicon starting materials (with different doping species) and made with different internal structures for the single microcells. In addition to the aforementioned technologies, we also developed particular types of SiPM structures that have position sensitivity (positions-sensitive SiPMs, PS-SiPM), specifically made with the so-called “linearly graded” (LG-SiPM) technology [18,19,20]. This is based on a position-weighted current partitioning approach, at the level of a single microcell (or few microcells).

The first version of LG-SiPM was based on FBK RGB-HD technology [18,19,20,21] and thus with peak sensitivity in the green. These detectors have been characterized with pulsed light and used in different scientific and medical applications [21], showing position reconstruction resolution below 250 μm on an 8 × 8 mm^2^ device area with four readout channels and minimal distortions. This technology was proven effective in the readout of segmented and monolithic LYSO crystals for PET application [21]. More recently we developed a second version of LG-SiPM with larger chip active areas (up to 10 × 10 mm^2^) and based on FBK NUV-HD technology, having a peak sensitivity around 420 nm and lower primary and correlated noise. This large-area SiPM detector, with good position sensitivity, might be very interesting in many applications where a reduced number of channels is needed, like MR-compatible PET [22,23], high-energy physics experiments, readout of time-projection chambers [24], readout of scintillating fibers for X-ray spectroscopy, and compact gamma and beta cameras [25].

In this work, we describe the LG-SiPM approach and the characterization results at the wafer and device levels of RGB-HD- and NUV-HD-based LG-SiPMs. Starting from the single large-area chip, we developed arrays of 2 × 2 and 3 × 3 elements, reaching very large sensitive areas of about 20 × 20 mm^2^ and 30 × 30 mm^2^. In such large-area arrays, we implemented a “smart-channel” configuration, which allowed us to have just six output channels for the 2 × 2 element tile and eight channels for the 3 × 3 element tile, still with a good position resolution below 0.5 mm. This solution provides great advantages compared to a similar detector configuration with single elements (i.e., read out individually) that would require hundreds of channels for the same spatial resolution. Instead, position-sensitive LG-SiPMs can be used to build larger arrays with a reduced number of channels and lower power consumption, which makes them a valid option for large-area systems.

## 2. Position-Sensitive SiPMs

In many applications, there is the need for high-sensitivity photodetectors that cover large areas but preserving the particle (or photon) arrival position information. This can be accomplished, for example, with arrays of SiPMs, each one read out by a dedicated front-end, either made by discrete components or by an ASIC chip. SiPM arrays are largely employed in applications like MR-compatible PET systems [26] or in instrumentation for space experiments [27]. However, in some cases, the system complexity, or simply the number of readout channels, needs to be reduced as much as possible, for example, in the case of small-animal PET or in compact detection modules for medical imaging, because of the small dimensions of the instrument [28].

As an alternative to SiPM arrays, position sensitivity over large areas can be obtained with position-sensitive SiPMs, large-area chips, or arrays of PS-SiPMs. In this case, each chip has four readout channels (top, bottom, left, and right position) as it is in other type of position-sensitive detectors like multi-anode PMT (MAPMT) [29] or resistive AC-coupled silicon detectors [30]. LG-SiPM chips have a large active area (e.g., 8 × 8 mm^2^) and a position resolution better than 0.5 mm × 0.5 mm. By contrast, in a SiPM array, the position resolution is comparable with the SiPM size, usually not smaller than 1 mm × 1 mm. It should be noted that the comparison of position resolution with other types of position-sensitive detectors is not straightforward due to the different internal gain, active area, and working conditions (e.g., light intensity or the number of photogenerated carriers). The results reported in the literature range from a few micrometers [30] up to ~0.5 mm [29].

Concerning position-sensitive SiPMs, different technological solutions have been developed over the years. For example, “sensitivity-encoding” SiPMs (SeSP) [31,32] and “interpolating” SiPMs [33,34] are based on scintillation light sharing among microcells that are physically connected to one of the four contacts, creating an amplitude and charge on the four signals that are proportional to the position of the flashing scintillator-array pixel. Another solution is based on a 2D resistive network that connects all the single elements of a SiPM array [35]. In this case, the signals are picked up at the four corners of the chip. Another development is the one where the large-area SiPMs have four contacts on a common-cathode region (on top) but with the microcells using an epitaxial quenching resistor (EQR), called tetra-lateral PS-SiPMs [36,37]. In such an approach, there are no quenching resistors on top of the microcells, because they are intrinsically integrated into the substrate of each SPAD; thus, it is possible to realize a common resistive cathode implantation, which also serves as a current divider for PS-SiPMs. 

### Linearly Graded (LG) SiPMs

**Figure 1:**
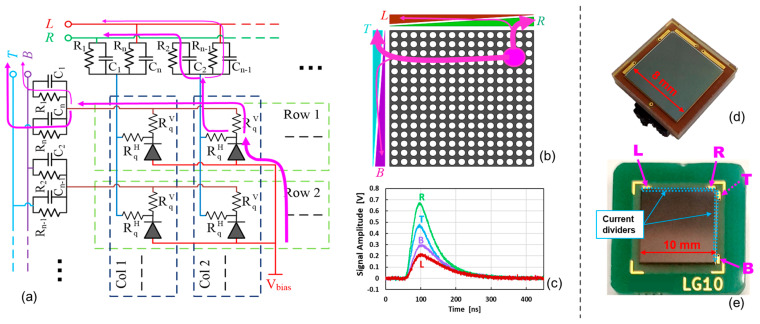
Schematic of the internal connections of SPADs, quenching resistors, and current dividers in an LG-SiPM (arrows represent the avalanche current paths) (**a**); representation of the current dividing approach for the left–right and top–bottom coordinates, depending on the position of the light flash on the active area of the SiPM (**b**); an example of the top, bottom, left, and right signals (after trans-impedance amplifier) (**c**); pictures of a packaged 8 × 8 mm^2^ (**d**) and 10 × 10 mm^2^ (**e**) LG-SiPMs.

In FBK, we developed a PS-SiPM technology called linearly graded (LG-SiPM). It is based on an avalanche-current spatially weighted-partitioning approach, at the level of a single microcell (i.e., SPAD) or a few microcells. The schematic representation of the internal structure is shown in Figure 1a, and its working principle is described in [18,19,20]. Each microcell of the SiPM is connected to two quenching resistors (instead of just one resistor as in standard SiPMs), one for the vertical current divider and one for the horizontal current divider, allowing us to obtain the vertical and horizontal positions of the light absorption. These two quenching resistors are identical; thus, as a first approximation, half of the current produced by SPADs is injected into each one. In the SiPM, each column (and each row) of microcells is connected to the same input of the current divider, i.e., to a couple of weighted resistors. In the current divider, the current is split between the two outputs, namely top–bottom for the vertical information and left–right for the horizontal information. The difference between the current flowing through the two arms (amplitude and total charge) changes linearly with the vertical (or horizontal) position, because the admittance of each resistor in the current divider varies linearly from one side to the opposite one, element by element, as schematized in Figure 1b,c.

We developed the first version of the LG-SiPM based on RGB-HD technology, with peak sensitivity in the green wavelength region, a chip size of ~8 mm (see Figure 1d), a breakdown voltage of ~28 V, and microcell pitch of 20 µm. A newer version was developed based on NUV-HD technology, with peak sensitivity at 420 nm, a chip size of ~10 mm (see Figure 1e), a breakdown voltage of ~32 V, and a microcell pitch of 25 µm.

In LG-SiPMs, the discretization for the position reconstruction is carried out almost at the single-cell level: typically, a few rows (or columns) of cells are connected to the same input of the current divider. The actual number (i.e., the discretization, in terms of the number of microcells) is a design choice, and it depends on the microcell size of the SiPM. Thanks to their working principle and their fine discretization, LG-SiPMs have the advantage of being highly scalable to large areas. It is possible to build either small chips, very large chips, or arrays of LG-SiPMs without degrading significantly the position resolution.

Having the amplitude of the four signals, it is possible to calculate the position of the interaction as the center of mass as follows:

$$Y=\frac{I_{T}-T_{B}}{I_{T}+I_{B}}\;\mathrm{and}\;X=\frac{I_{R}-I_{L}}{I_{R}+I_{L}}$$

where *I_T_*, *I_B_*, *I_R_*, and *I_L_* are the current at the top, bottom, right, and left contacts, respectively. The total number of detected photons, which is proportional to the total “energy” (*E*) of the particle causing the scintillating event, is simply calculated from the sum of the integral of all four current signals.

$$E\;\propto \;(I_{T}+I_{B}+I_{R}+I_{L})$$

## 3. Array of LG-SiPMs

Despite the LG-SiPM being already realized in large-area chips, if larger areas have to be covered, they can be arranged in arrays. For example, a 2 × 2 array of RGB-HD LG-SiPMs (nominal chip dimension of 8 × 8 mm^2^) has been used to build a high-resolution depth-encoding PET detector module [21]. In this case, the four SiPMs were read out independently, thus having a total number of 16 channels, covering a total sensitive area of 15.5 × 15.5 mm^2^, with a position resolution better than the pitch of the scintillator array, which was made by 30 × 30 elements of 0.45 mm × 0.45 mm each. 

With LG-SiPMs, it is possible to further reduce the number of channels when they are used in an array. Indeed, it is possible to physically connect some of the output signals (the closest ones) in a “smart-channel” configuration. In a 2 × 2 array, it is possible to connect the ”right” signal of both the left-placed chips and the “left” signals of the right-placed chips to have one common “horizontal-mid” (Hmid) signal. As a first approximation, this should not affect the position resolution. Indeed, depending on the interaction position, most of the time, there is a current signal from just one of the chips, e.g., the horizontal coordinate is extracted from the “left” and from the “H-mid” signals, as for a single LG-SiPM chip. Conversely, in the case of an illumination spot placed across two chips, there are signals from the “left”, “H-mid” and “right” terminals, where simply the H-mid signal is produced by summing together the currents from the two SiPM chips instead of just one, thus not changing the reconstruction procedure or the position resolution. In reality, it is possible that the parasitics added to the signal lines (e.g., higher capacitance and inductance) have a small effect on the signal shapes, thus affecting the reconstructed map shape and the position resolution. 

**Figure 2:**
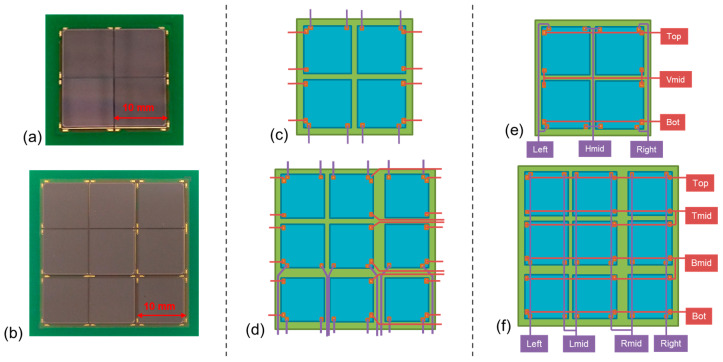
Picture of a 2 × 2 array of 10 × 10 mm^2^ LG-SIPMs (**a**) and a 3 × 3 array (**b**). The schematic representation of the number of output channels in the case of standard connections for 2 × 2 and 3 × 3 arrays (**c**,**d**) and in the case of “smart-channel” configuration (**e**,**f**).

With the reduced channel configuration, it is possible to obtain 6 output signals for a 2 × 2 array of LG-SiPM chips, 8 readout channels for a 3 × 3 array of chips (as shown in Figure 2), 10 readout channels for a 4 × 4 array, etc. The reduction in the number of channels is more and more important as the number of elements in the array increases. In the case of the 2 × 2 array, the vertical and horizontal coordinates can be obtained as follows:

$$Y=\frac{I_{T}-{\;I}_{B}}{I_{T}+I_{Vmid\;}+I_{B}}\;X=\frac{I_{R}-I_{L}}{I_{R}+I_{Hmid}+I_{L}}$$

whereas, for a 3 × 3 array, it can be calculated as follows:

$$Y=\frac{I_{T}+0.33\cdot I_{Tmid}-0.33\cdot I_{Bmid}-I_{B}}{I_{T}+I_{Tmid}+I_{Bmid}+I_{B}}\;Y=\frac{I_{R}+0.33\cdot I_{Rmid}-0.33\cdot I_{Lmid}-I_{L}}{I_{R}+I_{Rmid}+I_{Lmid}+I_{L}}$$

In this paper, we characterized the performance of an array of LG-SiPMs only when connected with the “smart-channel” configuration.

## 4. Performance Evaluation

### 4.1. Noise and Detention Efficiency

**Figure 3:**
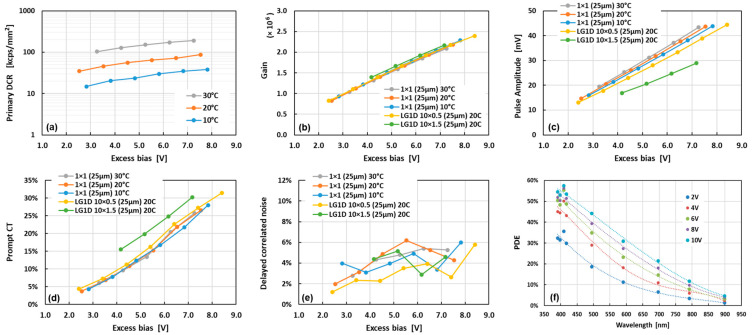
The primary dark count rate of NUV-HD-based LG-SiPMs, measured at different temperatures (**a**); microcell gain (**b**); SiPM pulse amplitude (using a trans-impedance amplifier) (**c**); prompt crosstalk probability (**d**); and delayed correlated noise, i.e., afterpulsing and delayed crosstalk (**e**) measured at different temperatures or for different LG-SiPM sizes; photon detection efficiency, measured at different wavelengths and excess biases (**f**).

The noise of the NUV-HD LG-SIPM was characterized at different temperatures and excess biases (i.e., the difference between the applied bias and the breakdown voltage). The method used in this paper is based on the analysis of trains of output pulses, identifying amplitudes and inter-times, and using these data to discriminate the primary noise events from the correlated noise events, as described in [1]. Because of the need to clearly separate and classify the pulses, the noise was measured on smaller-area SiPMs (i.e., 1 × 1 mm^2^), instead of the 10 × 10 mm^2^ LG-SiPMs, with the same microcell layout and taken from the same manufactured wafer of the larger devices. Indeed, because of the low-pass filtering effect in large-area SiPMs [1], the single-cell output pulse is usually not clearly distinguishable in these detectors. With 1 × 1 mm^2^ SIPMs, the main performance parameters are the same as those of large-area devices, except for the lower noise (absolute value but the same DCR per unit area) and pulse amplitude. As shown in Figure 3a, the primary noise (DCR) is about 60 kcps/mm^2^ (kilo-counts per second per square millimeter of active area) at 5 V of excess bias and 20 °C. The DCR halves (or doubles) roughly every 10 °C. 

The microcell gain is shown in Figure 3b, which is about 1.5 × 10^6^ at 5 V excess bias. In this plot, as in the others showing amplitude and correlated noise probabilities, we compared the results obtained with 1 × 1 mm^2^ SiPMs and with “1D” LG-SiPMs, with medium-sized area. Along with square-shaped “2D” LG-SiPMs with four output signals, we also produced rectangular-shaped “1D” LG-SiPMs, with active areas of 10 × 0.5 mm^2^ and 10 × 1.5 mm^2^, with just two output signals, providing the position information only for the long side of the rectangle. In this context, these devices were used because of their “medium-sized” area, to check the effect of the device size on the pulse shape and other functional parameters. In particular, we measured the pulses from the 1D LG-SiPMs with the two outputs shorted, thus working as a “regular” SiPM. We can see in Figure 3b that the gain is not changing with temperature or device size, as expected, since this quantity is mainly related to the capacitance of the microcells composing the SiPM [1]. Figure 3c shows the pulse amplitude obtained after the trans-impedance amplification of the output current (gain of 5000 V/A). We obtained around 29 mV at 5 V excess bias, and there was a small effect of temperature, probably due to variations in the recharge time constant [1]. Here, the effect of the SiPM size was more evident, with the pulse amplitude that decreased to ~26 mV for the 10 × 0.5 mm^2^ SiPM and ~20 mV for the 10 × 1.5 mm^2^ SiPM.

Figure 3d,e shows the prompt crosstalk probability and the delayed correlated noise probability (i.e., the sum of afterpulsing and delayed crosstalk probabilities). The former was ~13% at 5 V excess bias, not changing with temperature or SiPM size. The increment in the larger SiPM can be due to the effect of the increased primary DCR, producing a non-negligible probability of having two primary pulses almost superposed in time. The delayed correlated noise is generally small for NUV-HD technology, in the order of a few percent, and does not change with temperature (in the investigated range) or SiPM size.

We characterized the photon detection efficiency (PDE). The results are shown in Figure 3f for different wavelengths and excess biases. We used the method described in [38], based on an integrating sphere, a reference diode, and several pulsed LEDs. We measured a PDE value of around 50% at 6 V excess bias, at the peak (average value around 410 nm), which increased to 58% at higher excess bias. These values include the fill factor of the microcells, which is ~72% for a 25 µm cell pitch. Instead, it does not include the “chip-level FF”, i.e., the area lost on the border to host current partitioners and their interconnections to the four pads (since the PDE was measured on a 1 × 1 mm^2^ “standard” SIPM). However, on a large-area device, like a 10 × 10 mm^2^ device, the percentage of the active area was still high, above 92%.

### 4.2. Wafer-Level Pre-Selection 

Typically, SiPMs are always checked at the wafer level after manufacturing through forward and reverse current–voltage (IV) measurements, identifying the good and bad devices from breakdown voltage and other parameters. In the case of LG-SiPMs, this might not be enough, since the position resolution should also be checked. Indeed, it might be possible that, due to manufacturing-process-related issues, while a SiPM might be working properly in terms of dark current, breakdown voltage, etc., it can have problems in partitioning the avalanche current properly on the four outputs, thus preventing it from working as a position-sensitive detector. 

**Figure 4:**
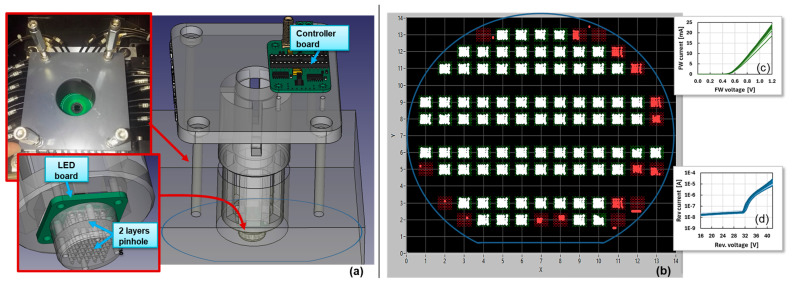
Representation of the custom LG-SiPM illuminator for automatic probers, including a 5 × 5 LED board and 2 layers of pinholes, placed in front of the LG-SiPM wafer to be measured (**a**); an example of measured wafer map of LG-SiPM reconstructed position (**b**); examples of forward and reverse current–voltage curves of 42 “good” 10 × 10 mm^2^ LG-SiPMs (**c**,**d**).

In order to be able to pre-screen LG-SiPMs, we developed a custom 5 × 5 LED illuminator, mounted as an add-on on the automatic prober. The prober included a customized probe card (with four probes) and a proper illumination opening, larger than the active area of the 10 × 10 mm^2^ SiPM. On top of the opening region, we placed the illuminator. This is sketched in Figure 4a. A controller board on 3D-printed support is used to turn on one LED at a time in the 5 × 5 array placed on a bottom board. In front of it, two arrays of metal pinholes of 0.4 mm diameter were used to reduce the illumination spot. 

During the wafer-level check procedure, we first performed the forward and reverse IV curves and then, at a fixed reverse bias voltage, we scanned the 25 illumination positions, acquiring the four output current levels and calculating the reconstructed positions. An example of a scanned wafer is shown in Figure 4b. Here, the reconstructed position array of the discarded SiPMs is marked in red and that of the good devices is shown in white. We can see that there might be cases where the reconstructed points are all compressed in one position (e.g., coordinate 3,2) or in one single row (e.g., coordinate 12,3), or where the reconstructed map is distorted toward just one half of the space (e.g., coordinate 9,13) or simply compressed in the center (e.g., coordinate 13,8).

### 4.3. Position Resolution of RGB-HD LG-SiPMs

**Figure 5:**
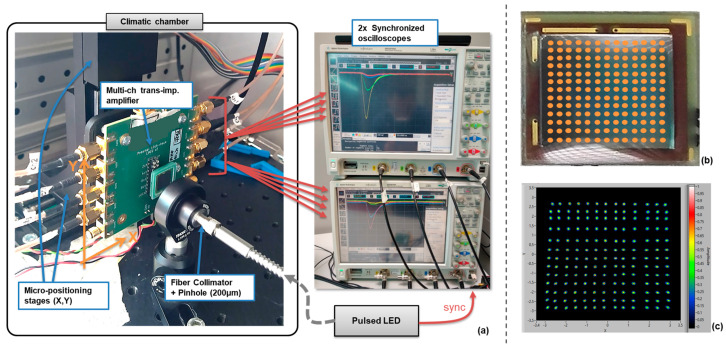
Position resolution measurement setup, including micropositioning stages, a multi-channel amplifier board, with output connected to 2 oscilloscopes (synchronized and triggered by the LED pulser) (**a**); an example of LG-SiPM scan: grid of illumination positions superposed with SiPM picture (step of 0.5 mm, range −3 to +3 mm) (**b**); and the relative reconstructed positions from SiPM channel signals (**c**).

After mounting the selected devices on PCB packages, we tested them in a setup like that shown in Figure 5a. It included a pulsed LED coupled to an optical fiber, which was placed inside a climatic chamber, where the light was collimated, and the spot was refined with a pinhole of 200 µm diameter. Inside the chamber, the SiPM was mounted on a custom multi-channel amplifier board (max 10 channels, trans-impedance amplification scheme), which was moved in front of the light spot through two-stage micropositioners. The outputs from the board were read out by two synchronized oscilloscopes (triggered by the LED pulser). 

An example of the scan matrix for the test of an LG-SiPM RGB-HD type (8 × 8 mm^2^ chip area) is shown in Figure 5b. The step was 0.5 mm, and the scanned area was between −3 mm and +3 mm in both directions. We used a 590 nm LED, with an intensity of ~350 detected photons per pulse, and the SiPM was kept at 20 °C with 4 V excess bias. The signals were integrated for 250 ns, including the peak and part of the recharge tail.

The map of the reconstructed positions (obtained after a linear stretching needed to meet the nominal scan area extension) is shown in Figure 5c. It can be seen that all the points are clearly distinguishable. There is a slight compression of the map toward the upper rows, but the calculated overall position resolution is σ = 0.054 ÷ 0.065 mm (average of the peak widths of all the scanned points). The average and standard deviation of the reconstructed positions were calculated considering around 1024 acquired groups of pulses per scan point. 

**Figure 6:**
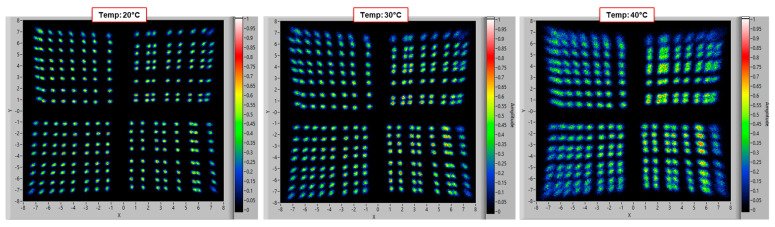
Map of reconstructed positions of a 2 × 2 array of LG-SiPM (RGB-HD-based), at different temperatures (20 °C, 30 °C, and 40 °C) (step of 1 mm). The effect of the SiPM noise (larger at higher temperatures) is visibly affecting the position resolution.

We also built a 2 × 2 array of such 8 × 8 mm^2^ SiPMs, using the “smart-channel” configuration (six total channels). We performed the same position resolution characterization described above and the results of the reconstructed position map are shown in Figure 6. We used a scan area of −7.5 mm to +7.5 mm, with a step of 1 mm, and we performed the test at different temperatures, namely 20 °C, 30 °C and 40 °C, to show the effect of primary noise generation (primary DCR) on the position resolution. We can see that all the positions in the map are clearly distinguishable, especially at low temperatures, demonstrating the feasibility of the “smart-channel” configuration. We can see also that there are some minor imperfections in some of the SiPM chips, for example, in the two SiPM chips on the right; some columns are closer to each other, and some are more distant. 

We can see that the points on the map become blurred when increasing the temperature to 40 °C. The estimated overall position resolution was σ = 0.140 ÷ 0.150 mm (the average of the peak width of all the scanned points) at 20 °C, but it increased to σ = 0.175 ÷ 0.195 mm at 30 °C and σ = 0.290 ÷ 0.340 mm at 40 °C. This effect is due to the primary noise being in the order of 300 kcps/mm^2^ at the lowest temperature and approximatively doubling every 10 °C of temperature. This is more relevant in an array of chips where more SiPMs are connected together because of the larger total area. The primary noise was smaller in the new NUV-HD-based LG-SiPM; thus, this effect was much reduced.

### 4.4. Position Resolution of NUV-HD LG-SiPMs

We measured the position resolution of the new NUV-HD-based LG-SiPM. The chip area was 10 × 10 mm^2^. We used the same setup shown in Figure 5a, but we used an LED at 410 nm (intensity of > 200 detected photons per pulse), given the peak sensitivity of this technology in the blue wavelength region. We performed a scan between −4.5 mm and +4.5 mm in both directions, with a step of 1 mm, the SiPM at 20 °C biased at 4.5 V excess bias, and an integration time of 200 ns.

**Figure 7:**
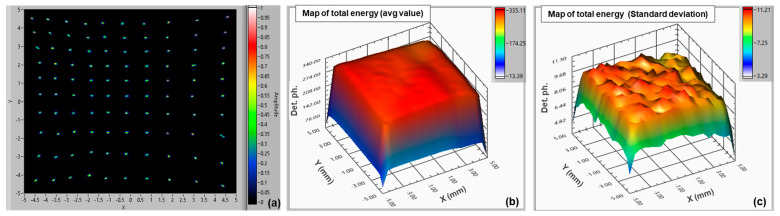
Map of reconstructed positions of a 10 × 10 mm^2^ LG-SiPM (NUV-HD based) (scan with step 1 mm) (**a**); map of energy resolution average value (**b**); and map of the standard deviation of “energy” resolution (**c**).

The map showing the reconstructed positions is presented in Figure 7a (after linear stretching to meet the nominal scan area extension). All the positions are clearly distinguishable. The calculated overall position resolution (averaged among all points in the map) was σ = 0.046 ÷ 0.056 mm. In these tests, we considered 512 groups of signals per scan point to obtain sufficient statistics on the reconstructed positions. A small pincushion effect can be observed on the map (also reported for other types of position-sensitive detectors [39,40]), which becomes slightly more evident when increasing the integration time of the signals. This might be related to parasitic inductances in such large-area SiPM chips, combined with the non-zero input impedance of readout electronics.

In Figure 7b,c, we also provide the map of the average and the standard deviation of the “energy” value, i.e., the sum of all the charges measured from the four SiPM signals, divided by the microcell gain, amplifier gain, elementary electron charge, and excess charge factor (ECF) [1]. This gives the number of detected photons in all the scan points. We can see that the sensitivity is uniform over the LG-SiPM active area. The standard deviation is in the order of 10, over an average level of ~330 detected photons.

### 4.5. Position Resolution of 2 × 2 and 3 × 3 NUV-HD LG-SiPM Arrays

We combined the 10 × 10 mm^2^ SiPM in 2 × 2 and 3 × 3 arrays, with the “smart-channel” configuration. We tested them with the same setup described in the previous section, scaled to larger areas. 

**Figure 8:**
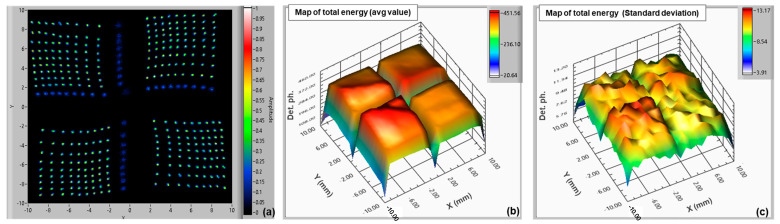
Map of reconstructed positions of a 2 × 2 array of 10 × 10 mm^2^ LG-SiPM (NUV-HD-based), with “smart-channel configuration” (scan with step 1 mm) (**a**); map of the energy resolution average value (**b**); map of the standard deviation of “energy” resolution (**c**).

For the 2 × 2 array of LG-SiPMs (with six output channels), we scanned over the range −10 mm to +10 mm, with a step of 1 mm, with the SiPM at 20 °C biased at 2.5 V excess bias. The map of the reconstructed positions is shown in Figure 8a (acquired with an integration time of 100 ns). All the points are clearly visible, except the ones at the edges of the chips (i.e., ±10 mm, not visible in the map) and also partly the ones at coordinates 0 mm, due to the light going outside the active area of the chips. This is clearly visible in the “energy” plot (Figure 8b). The separation between the chips is larger in this case (with respect to the ones in Figure 6), but there are fewer “imperfections” in the distribution of the points. Again, a small pincushion effect is also observed, indicating compression in the central part of the map with respect to the edges. The overall position resolution in this case was σ = 0.110 ÷ 0.115 mm, considering the whole active area of the tile, and thus almost 20 × 20 mm^2^. Figure 8b,c reports the “energy” maps, average value, and standard deviation (integration time 400 ns), giving information on the uniformity of the sensitivity. The active and dead areas of the 2 × 2 array are clearly visible, and despite some minor topography on the map, the uniformity is good, having a standard deviation in the order of ~12 photons on an average value of ~400 detected photons.

**Figure 9:**
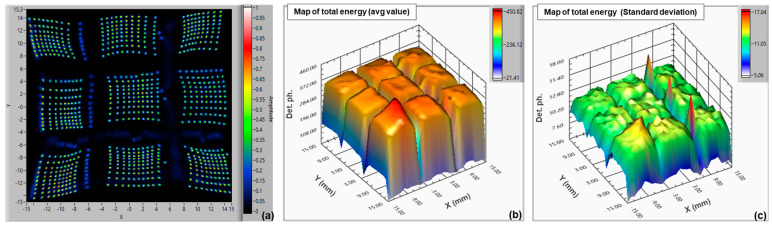
Map of reconstructed positions of a 3 × 3 array of 10 × 10 mm^2^ LG-SiPM (NUV-HD-based) (scan with step 1 mm, integration time 250 ns) (**a**). Map of energy resolution average value (**b**) and map of standard deviation (**c**) of “energy” resolution.

For the 3 × 3 array of LG-SiPMs (with eight output channels), we scanned over a range of −15 mm to +15 mm, with a step of 1 mm and the SiPM at 20 °C biased at 3.5 V excess bias. The map of the reconstructed positions is shown in Figure 9a (acquired with an integration time of 250 ns). All the points are clearly visible, except for the ones at the edges of the chips and in between the SiPM chips. The gap is larger between the second and third columns and between the second and third rows, because of the bonding wires, as shown in Figure 3b. The pincushion effect is more pronounced, with the maps of the LG-SiPM chips in the central row “compressed” toward the center with respect to the ones in the corners. As mentioned in the previous section, this effect might be enhanced by the “smart-channel” configuration, due to the higher parasitics, in particular the capacitance on the central signal lines (e.g., Tmid, Bmid, Rmid, and Lmid) having a large number of chips connected. It might also be due to the parasitic inductances in such large-area SiPM chips and the non-negligible input impedance of readout electronics. Further investigations are needed to better understand this feature.

The estimated average position resolution, excluding the regions outside the active area of the chips, was σ = 0.155 ÷ 0.180 mm. The measured “energy” maps, average value, and standard deviation (integration time 400 ns) are shown in Figure 9b,c, which give information on the uniformity of sensitivity (acquired with an integration time of 500 ns). As in the previous case, the standard deviation was in the order of ~12 on an average value of ~360 detected photons. 

**Figure 10:**
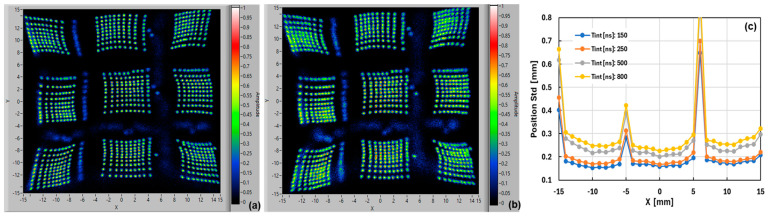
Map of reconstructed positions of a 3 × 3 array of 10 × 10 mm^2^ LG-SiPM (NUV-HD-based) (scan with step 1 mm), acquired with integration times of 500 ns (**a**) and 800 ns (**b**). Position resolution (standard deviation) as a function of the X coordinate at different integration times (**c**).

We also studied the effect of integration time on the position reconstruction performance. As an example, we compare the case of 250 ns, 500 ns, and 800 ns (Figure 9, Figure 10a and Figure 10b, respectively). The best position resolution was obtained with the smaller integration time; in particular, the best results were obtained when integration time was comparable with the SiPM pulse rise time plus recharge time constant. By further increasing the signal integration time, we improved the resolution in the “energy” signal, i.e., the overall number of photons detected, but the position resolution worsened. The reason is related to the lower “information content” of the SiPM signals, as described in [24]. In Figure 10c, we compare the measured position resolution (standard deviation) and the average of all the scanned rows, as a function of the vertical position (i.e., X coordinate), for four different integration times. As discussed above, it became significantly worse in the regions where the beam illuminated outside the chips’ active areas, whereas, considering only the sensitive areas, we obtained averages of 0.168 mm, 0.182 mm, 0.229 mm, and 0.255 mm, respectively, for integration times of 150 ns, 250 ns, 500 ns, and 800 ns. 

## 5. Conclusions

We described the working behavior, implementation, and performance characterization of the FBK position-sensitive “linearly graded” SiPM technology (LG-SiPM). Being based on avalanche-current spatially weighted partitioning, LG-SiPMs can be used to build large-area chips and arrays of chips, in a highly scalable approach. Moreover, with a “smart-channel” configuration, it is possible to group the different chip output channels in an array to have just six output channels in a 2 × 2 chip tile and eight channels in a 3 × 3 chip tile, thus greatly reducing readout electronic complexity and power consumption. We produced LG-SiPMs based on RGB-HD technology (green peak sensitivity) and newer ones based on NUV-HD technology (NUV peak sensitivity, with lower primary noise, i.e., in the order of 60 kcps/mm^2^ at 20 °C).

We developed a custom setup for prescreening these SiPMs at the wafer level, based on a 5 × 5 LED illuminator. We characterized the performance of LG-SiPMs with pulsed LED illumination, comparing single chips, 2 × 2 arrays, and 3 × 3 arrays (covering 10 × 10 mm^2^, 20 × 20 mm^2^, and >30 × 30 mm^2^ active area, respectively). We obtained position resolutions of about σ = 0.046 ÷ 0.056, σ = 0.110 ÷ 0.115 mm, and σ = 0.155 ÷ 0.180 mm, respectively. These values indicate good performance, considering the dimensions of large chips. The worsening of the position reconstruction when increasing the number of chips, and thus the dimension of the sensitive area, is likely related to the much larger parasitics (in particular, the capacitance of all the chips connected to one signal line in the “smart configuration”). We also characterized the effect of primary noise and integration time on the position resolution. 

Generally, these detectors provide great advantages in all applications requiring position resolution but with a small number of readout channels and reduced system complexity, for example, in small-animal PET or other types of compact gamma cameras.

## Data Availability

Data are contained within the article.
